# Supplementary material for: Exploring maintenance of physical activity behaviour change among people living with and beyond gastrointestinal cancer: a cross-sectional qualitative study and typology
Source: BMJ Open. 2020 Oct 29;10(10):e037136. doi: 10.1136/bmjopen-2020-037136 (PMC7597473; doi:10.1136/bmjopen-2020-037136)
Supplement: Supplementary data [file bmjopen-2020-037136supp002.pdf]

Appendix A – Interview guide

- What did you do as part of the [name physical activity programme]?

- How did you incorporate this into your day to day life?

- How are things now in relation to your physical activity habits?

- For those who describe increased/regular physical activity engagement:
    - What has helped you to make these changes?
    - Have there been any challenges to you staying active?
    - Why have you made these changes?
    - Has anyone else helped you to stay active?
    - Did you have to make changes to other parts of your life to incorporate more physical activity?
    - How does your activity impact on you? Prompt - How does it make you feel? Does it affect any other areas of your life?
    - In what way is physical activity important to your everyday life?

- For those who describe no change in physical activity levels:
    - Would you like to be doing anything differently?
    - If yes – what are the main things that prevent you from being more active?

- For those who describe an initial increase but then a decline in physical activity levels:
    - Why do you think your activity levels reduced after the trial?
    - Would you like to be doing anything differently?
    - If yes – what are the main things that prevent you from doing so?

- How do your physical activity habits compare to times before your cancer diagnosis?
